# Integrative Pharmacological and Computational Analysis of *Abelmoschus esculentus* Phytochemicals: Enzyme Inhibition, Molecular Docking, and Dynamics Simulation Against Key Antidiabetic Targets

**DOI:** 10.3390/life16030530

**Published:** 2026-03-23

**Authors:** Humera Banu, Eyad Al-Shammari, Fevzi Bardakci, Mitesh Patel, Mohd Adnan, Mohammad Idreesh Khan, Noor AlFahhad, Syed Amir Ashraf

**Affiliations:** 1 Department of Clinical Nutrition, College of Applied Medical Sciences, University of Ha’il, P.O. Box 2440, Ha’il 55473, Saudi Arabia; 2 Medical and Diagnostic Research Center, University of Ha’il, P.O. Box 2440, Ha’il 55473, Saudi Arabia; 3 Department of Biology, College of Science, University of Ha’il, P.O. Box 2440, Ha’il 55473, Saudi Arabia; 4 Department of Bioinformatics, Faculty of Engineering and Technology, Marwadi University, Rajkot 360003, Gujarat, India; 5 Department of Basic Health Sciences, College of Applied Medical Sciences, Qassim University, P.O. Box 6666, Buraydah 51452, Saudi Arabia; 6 Department of Family Medicine, College of Medicine, Qassim University, P.O. Box 6666, Buraydah 51452, Saudi Arabia

**Keywords:** *Abelmoschus esculentus*, diabetes mellitus, molecular docking, molecular dynamics simulation, alpha-Carotene, enzyme inhibition

## Abstract

The present work set out to examine the antidiabetic capacity of *Abelmoschus esculentus* (okra) fruit extract through a combined experimental and computational framework. Enzyme inhibition assays were carried out against four metabolic targets, and IC_50_ values stood at 7.66 ± 0.31 mg/mL for alpha-glucosidase, 5.21 ± 0.18 mg/mL for alpha-amylase, 2.11 ± 0.15 microg/mL for DPP-4, and 9.17 ± 0.54 mg/mL for pancreatic lipase. The extract showed moderate-to-weak activity relative to standard inhibitors acarbose, sitagliptin, and orlistat. Sixteen drug-like phytochemicals obtained from the IMPPAT 2.0 database were docked against the crystal structures of all four tested enzymes (PDB: 8CB1, 5E0F, 2ONC, 1LPB). Alpha-Carotene, Vitamin E, and Spiraeoside emerged as the top-ranked compounds across all targets, with alpha-Carotene recording the strongest binding affinity of −11.1 kcal/mol against pancreatic lipase, which was 4.2 kcal/mol more negative than the positive control orlistat (−6.9 kcal/mol). PLIP-based interaction profiling mapped out hydrogen bonds, hydrophobic contacts, pi-stacking, and salt bridges at the atomic level. Absorption, distribution, metabolism, and excretion (ADME) and toxicity screening of alpha-Carotene returned a favourable pharmacokinetic profile with predicted LD_50_ of 1510 mg/kg (Class 4) and inactivity across most toxicity endpoints. A 100 ns molecular dynamics simulation of the pancreatic lipase-alpha–Carotene complex, alongside the orlistat control, showed stable root mean square deviation (RMSD) (0.15–0.22 nm), a consistent Rg (~1.97 nm), and sustained hydrogen bonding throughout the trajectory. Free-energy landscape analysis revealed a well-defined single energy basin for alpha-Carotene, suggesting a thermodynamically stable binding conformation. These findings lay the molecular basis for using okra phytochemicals as adjunctive agents in diabetes management, though in vivo validation remains necessary.

## 1. Introduction

Diabetes mellitus is a long-lasting metabolic disorder characterised by either deficient insulin secretion or insulin resistance or sometimes both. Prolonged hyperglycemia, if left untreated, causes damage to almost every organ in the body, including the kidneys, eyes, nerves, and heart [1,2]. Over 500 million people worldwide suffer from diabetes, with the number expected to rise by 30% by 2045 [3]. Most of these cases are of type 2 diabetes mellitus (T2DM), which mainly involves insulin resistance, pancreatic β-cell dysfunction, as well as derangement of lipid and protein metabolism [4]. Excess glucose level makes cells produce more reactive oxygen species, leading to oxidative stress and thus damage at the molecular and cellular levels of the affected organs, such as the pancreas, liver, and kidney [5]. Drugs for the treatment of T2DM, such as oral hypoglycemic drugs and insulin, do have side effects (and patients are often non-compliant too), making it necessary to look for more natural and safer alternatives with less toxicity and multiple modes of therapeutic action [6].

Plant-based medicines are increasingly gaining recognition as safe and effective adjuvants in diabetes therapy. There are numerous medicinal plants that contain bioactive compounds capable of lowering glucose, scavenging free radicals, and improving lipid metabolism [7]. More studies are conducted on *Abelmoschus esculentus* L., (okra) because of its partly proven anti-diabetic effect, and it has received a lot of attention in the last few years. Okra belongs to the family Malvaceae, and its cultivation extent is quite huge in the tropical and sub-tropical regions of Africa, Asia, and the American continents [8]. This has been used among indigenous people not only for food but also for its medicinal value beforehand in the treatment of gastric irritation, obesity, and diabetes [9,10,11]. The various chemicals of okra that are responsible for its pharmacological properties are polysaccharides, flavonoids, and phenolic compounds with quercetin, catechin, and epigallocatechin [8,12]. These biologically active compounds are responsible for the antioxidative property and the regulation of the metabolism of glucose. Some research experiments agree that okra components protect against hyperglycemia [13,14] and hyperlipidemia [15,16] and also help reduce oxidative stress in diabetic models.

Many mechanisms are involved in okra’s efficacy as an anti-diabetic agent at the molecular level. Okra bioactives have been used to inhibit the activity of carbohydrate-hydrolysing enzymes like α-amylase and α-glucosidase that increase the digestion and absorption of glucose [17]. Apart from that, okra constituents are capable of down-regulating the level of dipeptidyl peptidase-4 (DPP-4), which deals with the breakdown of incretin hormones, which are the ones causing the secretion of insulin [2]. Furthermore, the insulin-degrading enzyme and tumour necrosis factor-α (TNF-α) levels in the liver are downregulated by okra, whereas the peroxisome proliferator-activated receptor (PPAR) signalling pathway in the pancreas gets modulated [18]. All these changes result in greater glucose uptake and insulin sensitivity. Okra fruit extracts have been seen lowering the level of total cholesterol and triglycerides in the blood, thus decreasing the risk of diabetic dyslipidemia [2,13]. Besides that, its antioxidant bioactives are capable of helping restore the redox homeostasis and protect pancreatic β-cells against oxidative stress. Such a wide array of effects makes okra an ideal natural anti-diabetic pharmaceutical candidate.

Several synthetic anti-diabetic drugs, such as metformin, sulfonylureas, and thiazolidinediones, are available on the market; however, the growing number of diabetic patients and the emergence of secondary complications call for more effective therapies [19,20]. Usually, long-term use of these drugs is accompanied by unwanted effects like gastrointestinal problems, hepatotoxicity, and cardiovascular issues [2,18,21]. The aforementioned concerns prompt the studies on plant-derived remedies since they are expected to provide good glycemic control with very few side effects. Several studies have recognised okra as one such excellent medicinal plant that can concurrently regulate various pathways of diabetes [21]. Phytochemical studies have shown the presence of α-carotene, lycopene, quercetin derivatives, catechin, and rutin, which have made okra have hypoglycemic and hypolipidemic activities [22,23,24]. Basically, these compounds interact with several molecular targets in the pathways of diabetes and cardiovascular diseases, thereby resulting in therapy with a very wide spectrum.

The present study aimed to bridge experimental enzyme-inhibition data with structure-based computational analysis of okra phytochemicals. Unlike previous reports that limited computational work to unrelated protein targets, this investigation performed molecular docking directly against the four enzymes tested in the laboratory assays: alpha-glucosidase, alpha-amylase, dipeptidyl peptidase-4 (DPP-4), and pancreatic lipase. Sixteen drug-like compounds from okra were screened against all four enzyme structures obtained from the Protein Data Bank. The top-ranked complex was then taken forward for 100 ns molecular dynamics simulation alongside its respective positive control drug to compare binding stability under physiological conditions. Absorption, Distribution, Metabolism, and Excretion (ADME) and toxicity profiling of the lead compound were also carried out to assess its pharmacokinetic suitability. This integrated approach provides a coherent experimental-to-computational pipeline for evaluating the antidiabetic capacity of okra at the molecular level.

## 2. Materials and Methods

### 2.1. Plant Material and Extract Preparation

Fresh okra (*Abelmoschus esculentus* L.) fruits were procured from a local agricultural market in Bengaluru, Karnataka, India. The fruits were washed thoroughly under running water to remove surface contaminants, then sliced into small pieces and shade-dried at room temperature for 7 days. Dried material was then ground into a fine powder using a mechanical grinder. The powdered sample (50 g) was subjected to aqueous extraction by soaking in 500 mL of distilled water at 60 °C for 6 hours with intermittent stirring. The resulting mixture was filtered through Whatman No. 1 filter paper, and the filtrate was concentrated using a rotary evaporator at 45 °C under reduced pressure. The dried residue was weighed, reconstituted in dimethyl sulfoxide (DMSO) to form a stock solution, and stored at −20 °C until use. Serial dilutions were prepared from this stock in DMSO to achieve concentrations of 10 mg/mL, 1 mg/mL, 100 µg/mL, 10 µg/mL, 1 µg/mL, and 0.1 µg/mL. All concentration values reported in this study refer to the mass of dried extract per unit volume of the final assay solution.

### 2.2. In Vitro Evaluation of Antidiabetic Enzyme Inhibitory Activity

The study aimed to assess the ability of *Abelmoschus esculentus* fruit extract to inhibit enzymes involved in diabetes and lipid metabolism. The enzymes tested were alpha-glucosidase, alpha-amylase, dipeptidyl peptidase-4 (DPP-4), and pancreatic lipase. Different drugs served as reference inhibitors in the comparison: acarbose for alpha-glucosidase and alpha-amylase, sitagliptin for DPP-4, and orlistat for pancreatic lipase. Three independent experiments were conducted for each enzyme, and the results were reported as the percentage inhibition of enzyme activity by the extract relative to the control.

### 2.3. α-Glucosidase Inhibitory Assay

Inhibition of the α-glucosidase enzyme was measured by a slightly modified commercial assay kit protocol. 10 µL of the extract or acarbose was added in a well of a clear 96-well microplate to 10 µL of assay buffer and 10 µL of α-glucosidase enzyme solution. The reaction mixture was brought to a total volume of 80 µL with a buffer, and the mixture was kept for 15–20 min at room temperature. 20 µL of p-nitrophenyl-α-D-glucopyranoside (PNPG) substrate was added, thus starting the reaction. A microplate reader (BioTek, Winooski, VT, USA) was used to measure the p-nitrophenol release at 410 nm. The percentage enzyme inhibition was derived from the absorbance values, whilst the IC_50_ values of both the extract and the reference compound were gained through non-linear regression analysis [25].

### 2.4. α-Amylase Inhibitory Assay

The α-amylase inhibitory assay was conducted based on a colourimetric method. A mixture of 50 µL of extract or acarbose and 50 µL of α-amylase solution was made in each well of a 96-well plate and kept at room temperature for 10 min. Then, 50 µL of soluble starch substrate was added to the reaction mixture, which was allowed to react for 3 min. The reaction was terminated by adding a 50 µL aliquot of 3,5-dinitrosalicylic acid (DNS) solution, followed by heating the mixture at 85–90 °C for 10 min. After the samples had cooled down, the absorbance was taken at 405 nm. Comparing with the control, the percentage inhibition of α-amylase was computed, and the obtained IC_50_ values were recorded [26].

### 2.5. Dipeptidyl Peptidase-4 (DPP-4) Inhibitory Assay

DPP-4 inhibition was measured as per the fluorescence inhibitor screening method. A black 96-well plate was used, and 50 µL of diluted DPP-4 enzyme solution was combined with 25 µL of the extract or sitagliptin as the reference compound. After pre-incubation of the mixture at 37 °C for 10 min, 25 µL of fluorogenic substrate was added to start the reaction and the mixture was incubated for 30 min at 37 °C. Setting the excitation and emission wavelengths at 360 nm and 460 nm, respectively, the fluorescence intensity was detected. The extent of inhibition of the enzyme was determined against the untreated control, wherein the IC_50_ values were also assessed [27].

### 2.6. Pancreatic Lipase Inhibitory Assay

The ability of the extract to inhibit lipase was assayed with p-nitrophenyl butyrate (PNB) as the chromogenic substrate. Each well was loaded with 50 µL of extract or orlistat combined with 20 µL of pancreatic lipase and 120 µL of Tris-HCl buffer (pH 7.4). The enzymatic mixture was kept at 37 °C for 25 min, and the reaction was started by the addition of 20 µL of the PNB substrate. To follow the release of p-nitrophenol, the absorbance at 450 nm was used. The percentage of the enzymatic activity was calculated by comparing the treated sample to the control, and the IC_50_ value was calculated from the inhibition curve [28].

### 2.7. Data Analysis

All enzyme inhibition tests were conducted three times, and the results were presented as mean ± standard deviation (SD). Percentage inhibition was determined using the formula:

$$\mathrm{Inhibition}\;(\%)=\left(\frac{A_{\mathrm{control}}-A_{\mathrm{sample}}}{A_{\mathrm{control}}}\right)\times 100$$

where $A_{\mathrm{control}}$ and $A_{\mathrm{sample}}$ represent the absorbance of control and test samples, respectively. The IC_50_ values were determined by fitting inhibition data to a sigmoidal dose–response curve using non-linear regression analysis. One-way analysis of variance (ANOVA) followed by Tukey’s post hoc test was used to compare the mean inhibition percentages of the okra extract with those of the respective standard inhibitors at each concentration. A *p*-value below 0.05 was taken as the threshold for statistical difference. All statistical analyses were performed using GraphPad Prism version 9.0.

### 2.8. Ligand Retrieval and Preparation

Phytoconstituents from *Abelmoschus esculentus* (okra) were sourced from the Indian Medicinal Plants, Phytochemistry and Therapeutics 2.0 (IMPPAT 2.0) database (https://cb.imsc.res.in/imppat/, accessed 15 October 2025). IMPPAT 2.0 is a manually curated digital repository that provides information on more than 4000 Indian medicinal plants, their phytochemicals, and the therapeutic relevance of these compounds, either experimentally or computationally derived [29]. For this research, the phytochemicals of A. esculentus as reported in the database were obtained in both 2D and 3D structural formats (SDF and MOL2). The compounds were then put through a first screening based on their drug-likeness, Lipinski’s rule of five, and ADMET properties to confirm their pharmacological potential for docking studies. Before docking, the selected ligand structures were energy-minimised and converted to Protein Data Bank, Partial Charge (Q), & Atom Type (T) (PDBQT) format using Open Babel. This step helped achieve geometric optimisation and eliminate redundant conformers for stable ligand poses in binding-site simulations.

### 2.9. Identification of Drug-like Compounds

Drug-like phytochemicals of *Abelmoschus esculentus* from the IMPPAT 2.0 database were filtered using ChemBioServer 2.0 (https://chembioserver.vi-seem.eu/, accessed 15 October 2025) to check their physicochemical suitability for pharmacological applications [30]. Each compound was evaluated against the Lipinski, Veber, and Ghose drug-likeness criteria. Compounds that fulfilled the Lipinski criteria of molecular weight below 500 Da, hydrogen bond donors of 5 or fewer, hydrogen bond acceptors of 10 or fewer, and logP of 5 or below; the Veber rule constraints of polar surface area of 140 square angstroms or less and rotatable bonds of 10 or fewer; and the Ghose filter limits of total atoms between 20 and 70 with molar refractivity between 40 and 130 were chosen. Compounds that satisfied all three rules in their entirety were treated as drug-like candidates. Those compounds that passed two of the three filters with only minor deviations in logP were retained as nutraceutical-type leads rather than strict drug candidates, given that dietary terpenoids and tocopherols follow different absorption and distribution pathways from typical oral drugs [30].

### 2.10. Molecular Docking and Interaction Analysis

Molecular docking was performed to assess the binding affinity of the screened okra phytochemicals to the four enzymes tested experimentally. The three-dimensional structures of all four target proteins were obtained from the RCSB Protein Data Bank (https://www.rcsb.org/, accessed 18 October 2025). Alpha-amylase was retrieved under PDB ID 5E0F, alpha-glucosidase under 8CB1, DPP-4 under 2ONC, and pancreatic lipase under 1LPB. Preparation of each protein structure was performed using AutoDock Tools version 1.5.7. Water molecules present in the crystal structures were removed at this stage. Co-crystallised ligands were also stripped out. Polar hydrogen atoms were then added to each receptor. Kollman charges were assigned to make the receptors ready for docking calculations. The prepared receptor files were saved in PDBQT format.

PyRx 0.9 served as the platform for docking simulations, running the AutoDock Vina engine in the background [31]. A blind docking approach was adopted for this study, allowing ligands to explore all potential binding pockets present on the protein surface. Grid box coordinates and dimensions for each protein were defined as follows: alpha-amylase (5E0F) centre at X = −8.21, Y = 21.50, Z = −18.80 with dimensions 56.71 × 73.26 × 54.87 angstroms; alpha-glucosidase (8CB1) centre at X = 1.13, Y = −24.19, Z = 91.45 with dimensions 83.08 × 62.87 × 76.89 angstroms; DPP-4 (2ONC) centre at X = 6.30, Y = −10.35, Z = 20.95 with dimensions 73.83 × 78.23 × 81.79 angstroms; pancreatic lipase (1LPB) centre at X = −1.58, Y = 33.19, Z = 41.97 with dimensions 77.65 × 56.10 × 59.49 angstroms. The exhaustiveness parameter was set to 8, balancing computational efficiency with search depth. Binding affinities were calculated in kcal/mol, and docking poses with the lowest binding energy and RMSD values near zero were selected for interaction analysis [31]. Positive control drugs were also docked under the same conditions: orlistat for pancreatic lipase (−6.9 kcal/mol), sitagliptin for DPP-4 (−9.1 kcal/mol), acarbose for alpha-glucosidase (−7.2 kcal/mol), and miglitol for alpha-amylase (−5.7 kcal/mol). These values served as benchmarks for comparing the phytochemical binding strengths [31].

### 2.11. Interaction Visualisation

Post-docking, the selected receptor–ligand complexes were analysed and visualised using BIOVIA Discovery Studio Visualizer (v2020.1) [31]. Hydrogen bonds formed between ligand atoms and protein residues were mapped out. Hydrophobic contacts and pi-related interactions were also identified at this stage. Two-dimensional interaction diagrams and three-dimensional structural representations were generated for inclusion in the Section 3.

### 2.12. Protein–Ligand Interaction Profiling

To gain a deeper look at the molecular interactions between the docked phytochemicals and the four target enzymes, the Protein-Ligand Interaction Profiler (PLIP) web server (https://plip-tool.biotec.tu-dresden.de/plip-web/plip/index, accessed 20 October 2025) was used. The best docking conformations obtained from AutoDock Vina were given to the PLIP interface in .pdb format for interaction profiling. The tool identified different types of interactions and grouped them into hydrogen bonds, hydrophobic contacts, pi-pi stacking, pi-cation interactions, salt bridges, halogen bonds, and water-mediated contacts. Interaction reports and schematic visuals were examined to identify the key residues involved in ligand binding and to assess the three-dimensional arrangement of these interactions within the active sites [32].

### 2.13. ADME and Toxicity Prediction

The pharmacokinetic and toxicological profile of the top-ranked compound (alpha-Carotene) was predicted using the SwissADME web server (http://www.swissadme.ch/, accessed 8 September 2025) and ProTox-II (https://tox-new.charite.de/protox_II/, accessed 22 October 2025). SwissADME was used to evaluate physicochemical properties and drug-likeness parameters (Lipinski, Veber, Egan, Ghose), as well as pharmacokinetic descriptors, including gastrointestinal absorption, blood–brain barrier permeability, P-gp substrate status, and cytochrome P450 inhibition profiles. ProTox-II served as the tool for predicting organ-level toxicity (hepatotoxicity, nephrotoxicity, cardiotoxicity, respiratory toxicity), toxicity endpoints (carcinogenicity, mutagenicity, cytotoxicity, immunotoxicity), Tox21 nuclear receptor signalling pathway disruption, stress response pathways, and molecular initiating events. The predicted LD_50_ value and toxicity class were also recorded [31].

### 2.14. Molecular Dynamics Simulation

Molecular dynamics (MD) simulations were carried out on the top-ranked protein–ligand complex, pancreatic lipase (1LPB) bound to alpha-Carotene (binding affinity: −11.1 kcal/mol), to assess its stability, conformational behaviour, and dynamic properties under physiological conditions. The same simulation protocol was applied to the positive control complex, pancreatic lipase bound to orlistat (−6.9 kcal/mol), to allow direct comparison of binding stability. Both complexes were subjected to 100 ns all-atom MD simulations using GROMACS 2023.3 [33].

The protein structures were parameterised using the CHARMM36 force field, whereas ligand topologies and partial charges were generated using CHARMM General Force Field (CGenFF) parameters. The individual systems were placed in a triclinic box and solvated with TIP3P water molecules. The distance between the protein surface and the box edge was at least 1.0 nm to avoid misinterpreted interactions. Appropriate counterions (Na^+^/Cl^−^) were added to achieve system neutrality. First, the energy minimisation step was implemented by the steepest descent method to eliminate existing strain and energetically unfavourable contacts. When the convergence criterion of 10^−6^ kJ/mol/nm was met, the minimisation process was stopped, allowing for the equilibration step [34].

Equilibration in the NVT ensemble (constant Number, Volume, and Temperature) was carried out for 100 ps at 310 K with a velocity-rescaling thermostat. Subsequent equilibration in the NPT ensemble (constant Number, Pressure, and Temperature) was run for 100 ps at 1 bar pressure using the Parrinello-Rahman barostat. After equilibration, the production run was performed for 100 ns without restraints. A 2 fs time step was used under periodic boundary conditions. Long-range electrostatic interactions were calculated by the Particle Mesh Ewald (PME) method; short-range nonbonded interactions were handled by the Verlet cutoff scheme with a 1.0 nm cutoff radius. The LINCS algorithm was used to constrain all hydrogen covalent bonds. The trajectory was saved every 2 ps. Post-simulation analyses included root mean square deviation (RMSD) of the backbone, root mean square fluctuation (RMSF) of individual residues, radius of gyration (Rg) for compactness, solvent-accessible surface area (SASA) for surface exposure, and hydrogen bond counts between the ligand and the protein over time. Visualisation of all MD trajectories was performed with VMD 1.9.3 and BIOVIA Discovery Studio Visualizer, whereas Xmgrace v5.1.25 and Matplotlib v3.10 were used to generate plots [35].

### 2.15. Free Energy Landscape and PCA

Principal component analysis (PCA) was carried out on the 100 ns MD simulation trajectories of both the pancreatic lipase–alpha-Carotene and pancreatic lipase–orlistat complexes to map their conformational dynamics. A covariance matrix of atomic positional fluctuations was created after the removal of translational and rotational motions. It was then diagonalised to obtain eigenvectors and their eigenvalues. The first two principal components (PC1 and PC2), which together account for the largest portion of atomic motion, were chosen to depict the essential dynamics of the systems [36]. The Free Energy Landscape (FEL) was plotted using PC1 and PC2 as reaction coordinates, and the Gibbs free energy surface was calculated from the Boltzmann distribution (delta-G = −RT ln P). Deep, sharply defined energy minima corresponded to stable conformational ensembles, while the transitions between local minima illustrated how the proteins adjust their conformations upon ligand binding [36].

## 3. Results

### 3.1. α-Glucosidase Inhibitory Activity

**Figure 1:**
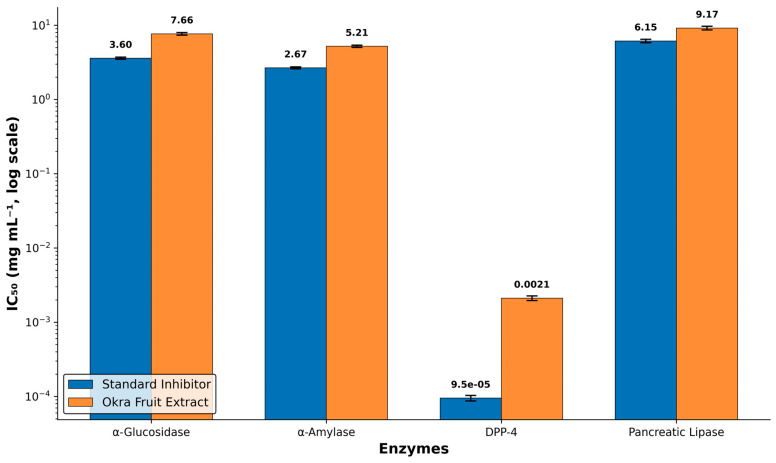
Comparative IC_50_ values of okra fruit extract and standard enzyme inhibitors. Bars show the mean IC_50_ (mg/mL, log scale) with error bars representing standard deviation from three independent experiments. Standard inhibitors are shown in blue; okra fruit extract in orange.

Okra fruit extract inhibited the enzyme alpha-glucosidase in a concentration-dependent manner. The IC_50_ value for the extract stood at 7.66 ± 0.31 mg/mL, whereas acarbose had an IC_50_ of 3.60 ± 0.12 mg/mL (Table 1; Figure 1). The extract was roughly 2.1-fold less potent than the standard inhibitor. While this level of inhibition is moderate and falls short of the reference drug, the presence of active principles in the crude extract capable of delaying carbohydrate breakdown is worth noting. Fractionation of the extract may help isolate the specific components responsible for this activity and could yield more concentrated inhibitory fractions.

### 3.2. α-Amylase Inhibitory Activity

The okra fruit extract showed a gradual, concentration-dependent inhibitory effect on alpha-amylase, with an IC_50_ of 5.21 ± 0.18 mg/mL, compared with 2.67 ± 0.08 mg/mL for acarbose (Table 1; Figure 1). The extract was about 1.95-fold weaker than the standard. From a practical standpoint, moderate alpha-amylase inhibition is often better tolerated physiologically, as very high levels of inhibition are usually associated with gastrointestinal discomfort. The moderate nature of this activity may be an advantage for long-term dietary supplementation rather than acute pharmacological intervention.

### 3.3. Dipeptidyl Peptidase-4 (DPP-4) Inhibitory Activity

The okra fruit extract had an IC50 of 2.11 ± 0.15 microg/mL for DPP-4, whereas the standard drug, sitagliptin, showed a much lower IC_50_ of 0.095 ± 0.008 microg/mL (Table 1; Figure 1). The extract was approximately 22-fold weaker than sitagliptin, making it the least active of the four enzymes tested. The large potency gap between the crude extract and a highly optimised synthetic drug is not unexpected, and the result should be understood in that light. A crude aqueous extract cannot be expected to match the potency of a drug that underwent years of medicinal chemistry optimisation. Even so, the fact that an unfractionated plant extract can modulate DPP-4 activity at the microgram level remains of interest for nutraceutical development rather than direct pharmaceutical application.

### 3.4. Pancreatic Lipase Inhibitory Activity

The okra extract gave an IC_50_ of 9.17 ± 0.54 mg/mL for pancreatic lipase, compared with 6.15 ± 0.32 mg/mL for orlistat (Table 1; Figure 1). The extract was about 1.49-fold less potent than the reference drug. Among the four enzymes tested, the smallest fold-difference between the extract and the standard was observed here, suggesting a more similar inhibitory capacity. Given that pancreatic lipase inhibition reduces dietary fat absorption and helps manage dyslipidemia in diabetic patients, the modest activity of the crude extract could still have a supporting role in metabolic regulation when consumed as part of a dietary regimen.

### 3.5. Molecular Docking

**Figure 2:**
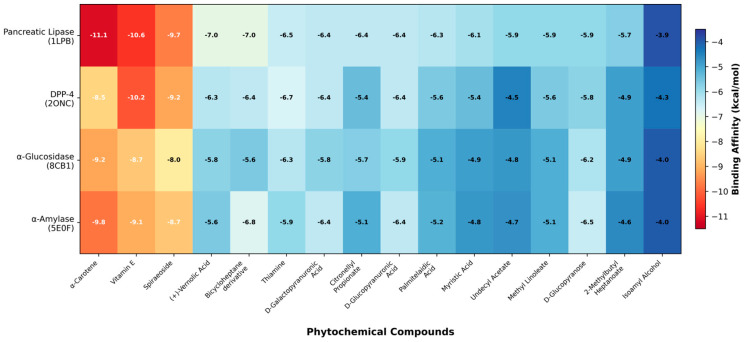
Heatmap of molecular docking binding affinities (kcal/mol) of sixteen okra phytochemicals against four antidiabetic enzyme targets. Blue tones represent stronger (more negative) binding; red tones represent weaker binding.

Docking scores for all sixteen okra phytochemicals against the four enzyme targets are presented in Table 2 and Table 3 and Figure 2. The three compounds with the strongest binding affinities across all targets were alpha-Carotene, Vitamin E, and Spiraeoside. Alpha-Carotene recorded the most negative binding energy of −11.1 kcal/mol against pancreatic lipase (1LPB), followed by −9.8 kcal/mol against alpha-amylase (5E0F), −9.2 kcal/mol against alpha-glucosidase (8CB1), and −8.5 kcal/mol against DPP-4 (2ONC). Vitamin E came in second across most targets, with binding energies ranging from −10.6 kcal/mol (pancreatic lipase) to −8.7 kcal/mol (alpha-glucosidase). Spiraeoside ranked third, with values between −9.7 kcal/mol (pancreatic lipase) and −8.0 kcal/mol (alpha-glucosidase).

Compared with the positive control drugs, alpha-Carotene outperformed orlistat by 4.2 kcal/mol for pancreatic lipase and was stronger than acarbose by 2.0 kcal/mol for alpha-glucosidase. Against alpha-amylase, alpha-Carotene outperformed miglitol by 4.1 kcal/mol. For DPP-4, sitagliptin (−9.1 kcal/mol) retained an edge over alpha-Carotene (−8.5 kcal/mol). Overall, the data point to alpha-Carotene and Vitamin E as the most promising binders among the okra compounds, with pancreatic lipase being the target for which the phytochemical binding advantage over the standard drug was largest.

### 3.6. Interaction Visualisation

**Figure 3:**
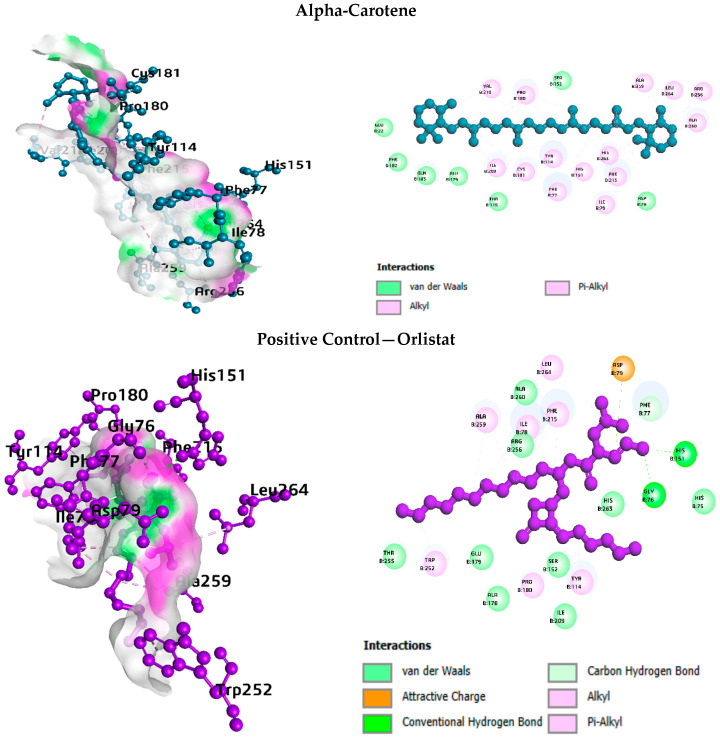
Interaction analysis of pancreatic lipase with top phytocompound and the standard drug.

**Figure 4:**
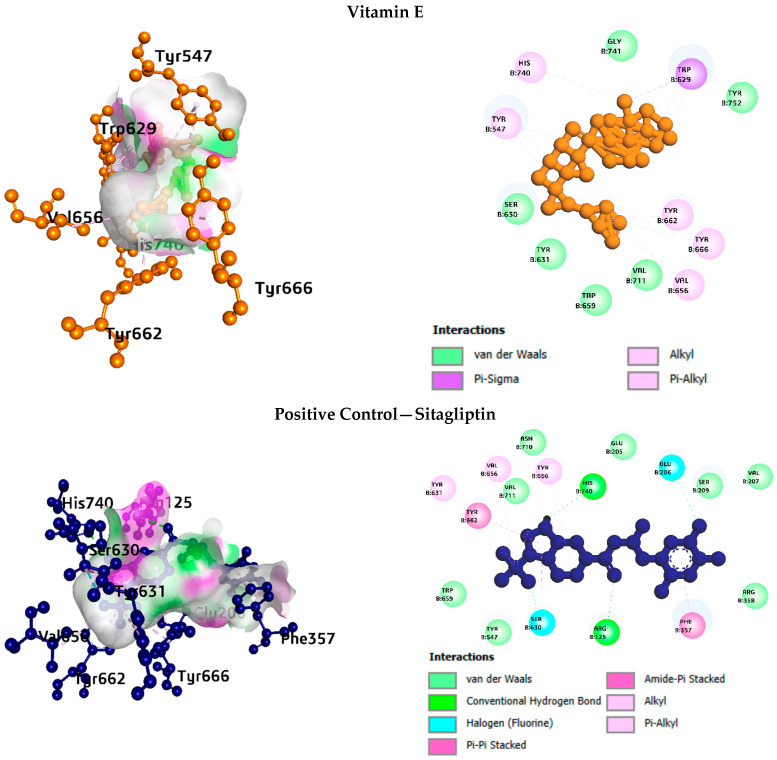
Interaction analysis of DPP4 with the top phytocompound and the standard drug.

**Figure 5:**
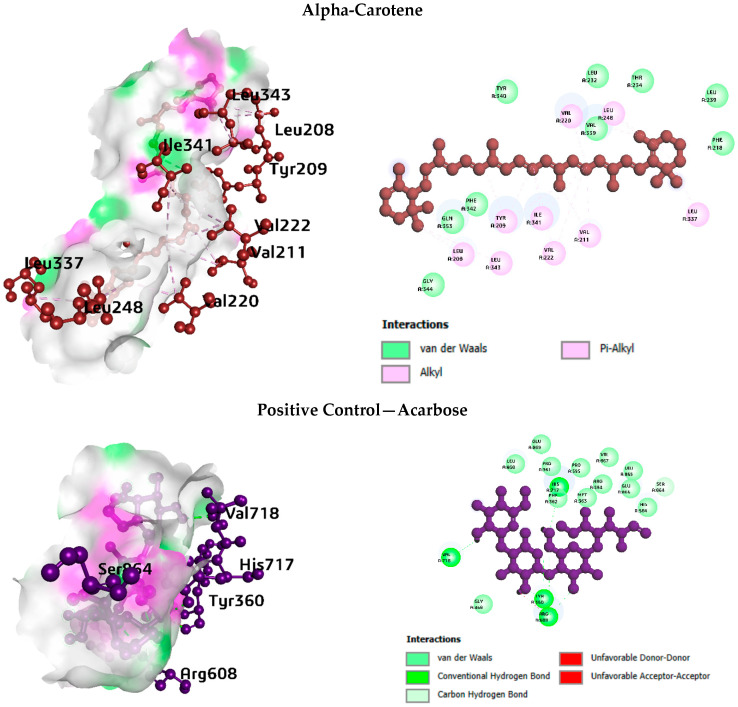
Interaction analysis of alpha-glucosidase with the top phytocompound and the standard drug.

**Figure 6:**
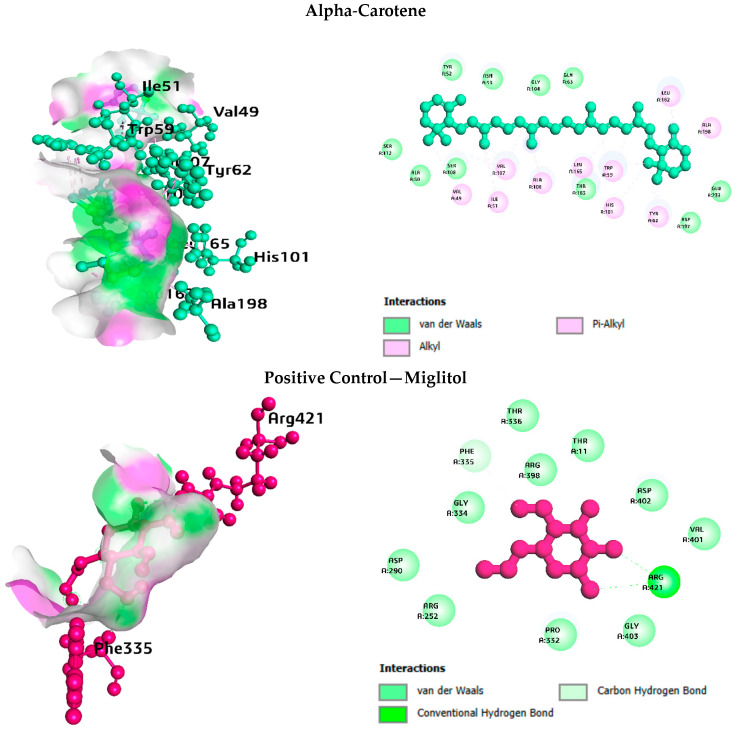
Interaction analysis of alpha-amylase with the top phytocompound and the standard drug.

3D binding poses and 2D interaction diagrams for the top-ranked compounds and their respective positive controls against each enzyme target are presented in Figure 3, Figure 4, Figure 5 and Figure 6.

a. Interaction analysis with pancreatic lipase

For pancreatic lipase (Figure 3), alpha-Carotene occupied a hydrophobic cleft lined by Tyr114, Phe77, Phe215, Pro180, Ile209, and Leu264 residues. Vitamin E adopted a similar orientation in the same pocket, with additional hydrogen bonds to Arg256. Spiraeoside formed multiple hydrogen bonds with Gly76, Asp79, His151, Ser152, and Arg256, along with pi-stacking with Tyr114 and a salt bridge with His263, pointing to a mixed polar and nonpolar binding mode. The positive control, orlistat, showed extensive hydrophobic contacts, complemented by hydrogen bonds to Gly76, Phe77, His151, and Ser152, and a salt bridge with Asp79.

b. Interaction analysis with DPP4

For DPP-4 (Figure 4), Vitamin E interacted primarily through hydrophobic contacts with Tyr547, Trp629, Tyr631, Trp659, and Tyr662, along with a hydrogen bond to Gly741. Spiraeoside formed twelve hydrogen bonds involving Arg125, Glu206, Ser209, Phe357, Arg358, Glu361, and Tyr666, making it the most polar binder for this target. Alpha-Carotene engaged eight hydrophobic contacts at the Phe357, Tyr547, and Trp629 residues. Sitagliptin, the positive control, combined hydrophobic interactions with hydrogen bonds to Asn710 and a halogen bond to Val207.

c. Interaction analysis with alpha-glucosidase

For alpha-glucosidase (Figure 5), alpha-Carotene formed fourteen hydrophobic contacts spanning Tyr209, Val211, Val220, Val222, Leu248, Leu337, Ile341, Phe342, and Leu343. Vitamin E occupied a distinct pocket engaging Trp376, Trp481, Phe525, Trp613, and Phe649. Spiraeoside formed four hydrogen bonds with Leu355, Met363, and Arg608, plus a salt bridge with His584. Acarbose, the positive control, formed nine hydrogen bonds and two salt bridges, confirming its strong polar binding character.

d. Interaction analysis with alpha-amylase

For alpha-amylase (Figure 6), alpha-Carotene formed thirteen hydrophobic contacts with Val49, Ile51, Tyr52, Trp59, Tyr62, Ala106, Val107, Leu162, Leu165, and Ala198. Vitamin E also favoured hydrophobic interactions with Trp58, Trp59, Tyr62, and Leu165, and formed one hydrogen bond with Gln63. Spiraeoside showed a mixed profile with five hydrophobic contacts and six hydrogen bonds to Gln63, His101, Asp197, Ala198, Asp300, and His305. Miglitol, the positive control, relied mainly on hydrogen bonds (thirteen) with minimal hydrophobic contribution.

### 3.7. Post-Docking Interaction Profiling

**Figure 7:**
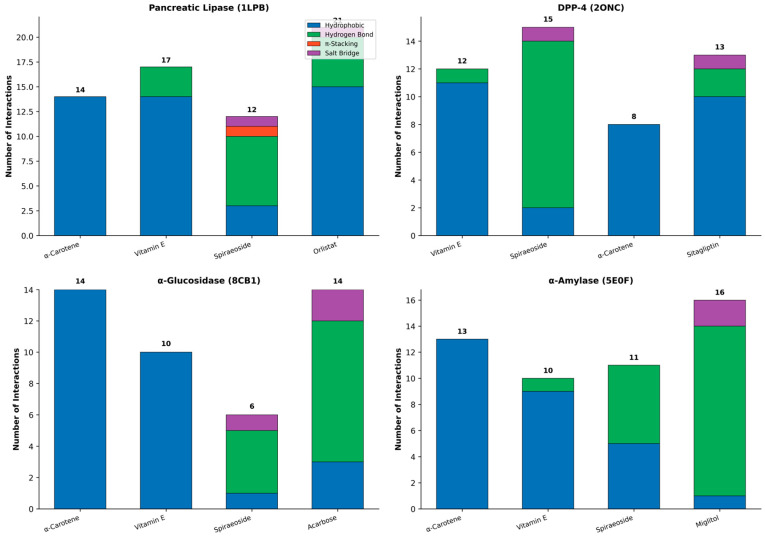
Summary of non-covalent interaction counts from PLIP analysis for the top three phytochemicals and respective positive controls across all four enzyme targets. Interaction types include hydrophobic (blue), hydrogen bonds (green), pi-stacking (red), and salt bridges (purple).

The PLIP analysis at the atomic level provided interaction details that are consistent with the docking affinity results (Table 4, Table 5, Table 6 and Table 7; Figure 7). Across all four enzymes, alpha-Carotene and Vitamin E relied mainly on hydrophobic contacts owing to their lipophilic chemical nature. Alpha-Carotene formed 14 hydrophobic interactions with pancreatic lipase, 8 with DPP-4, 14 with alpha-glucosidase, and 13 with alpha-amylase. No hydrogen bonds were recorded for alpha-Carotene at any of the four targets, which is expected given its fully hydrocarbon structure with no heteroatoms available for polar bonding. Spiraeoside displayed the most balanced interaction profile across the four enzymes. At pancreatic lipase, it formed seven hydrogen bonds (Gly76, Asp79, His151, Ser152, Arg256), one pi-stacking (Tyr114), and one salt bridge (His263). At DPP-4, twelve hydrogen bonds were mapped, making it the strongest polar binder for that target. At alpha-glucosidase and alpha-amylase, Spiraeoside combined hydrophobic contacts with four and six hydrogen bonds, respectively. The positive control drugs showed interaction profiles consistent with their known pharmacology: orlistat formed a mix of hydrophobic and hydrogen bond contacts at the pancreatic lipase active site, while acarbose and miglitol relied heavily on hydrogen bonds at their respective enzyme targets.

### 3.8. Pharmacokinetic and Toxicity Profile of Alpha-Carotene

**Figure 8:**
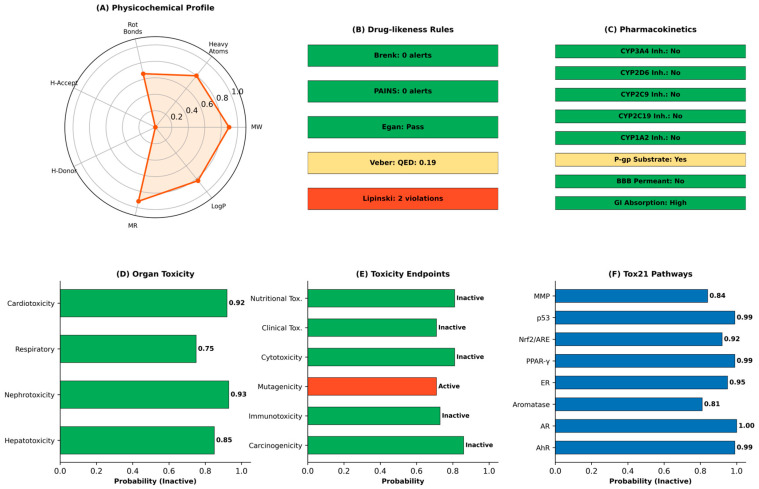
Combined ADME and toxicity profile of α-Carotene. (**A**) Radar plot of normalised physicochemical properties. (**B**) Drug-likeness rule compliance: green = pass, yellow = caution, red = violation. (**C**) Pharmacokinetic parameters: green = favourable, yellow = requires attention. (**D**) Organ toxicity predictions with probability scores (all inactive). (**E**) Toxicity endpoint predictions: green = inactive, red = active (mutagenicity). (**F**) Tox21 nuclear receptor and stress response pathway predictions with probability scores (all inactive).

The ADME profile of alpha-Carotene (C40H56, MW 536.89 g/mol) showed high gastrointestinal absorption with no blood–brain barrier permeation (Table 8; Figure 8). The compound was predicted to be a P-gp substrate, which may affect its oral bioavailability through P-gp-mediated efflux. No inhibition was predicted for any of the five major cytochrome P450 isoforms (CYP1A2, CYP2C19, CYP2C9, CYP2D6, CYP3A4), pointing to a low risk of drug–drug interactions. Drug-likeness assessment returned two Lipinski violations, which is expected for a large terpenoid compound. The QED weighted score stood at 0.19, and the compound passed Egan and PAINS filters with zero alerts.

Toxicity prediction using ProTox-II yielded a predicted LD_50_ of 1510 mg/kg, placing alpha-Carotene in toxicity Class 4 (harmful if swallowed; 300 < LD_50_ < 2000). Organ-level predictions returned inactive status for hepatotoxicity (probability 0.85), nephrotoxicity (0.93), respiratory toxicity (0.75), and cardiotoxicity (0.92) (Table 9). Among the toxicity endpoints, the only active prediction was for mutagenicity (probability 0.71). This finding warrants further investigation through the Ames test or in vivo genotoxicity assays before any clinical consideration. All Tox21 nuclear receptor signalling pathways and stress response pathways returned inactive predictions with high probability values ranging from 0.81 to 1.00.

### 3.9. Molecular Dynamics Simulation Analysis

**Figure 9:**
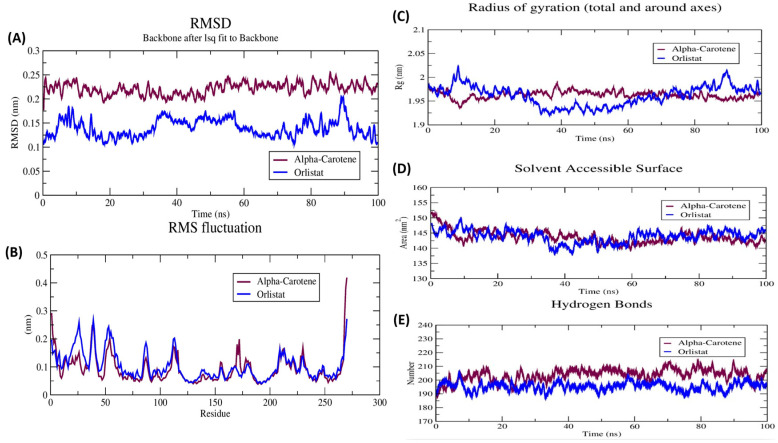
Molecular dynamics simulation analysis of pancreatic lipase–α-Carotene (maroon) and pancreatic lipase–orlistat (blue) complexes over 100 ns. (**A**) Backbone RMSD profile showing structural convergence after ~20 ns for both complexes; α-Carotene: 0.15–0.22 nm, orlistat: 0.10–0.25 nm. (**B**) RMSF per residue showing localised flexibility at surface loops and terminal regions; a pronounced peak at residues 270–280 (lid domain) in the α-Carotene complex. (**C**) Radius of gyration (~1.95–2.00 nm) confirming no unfolding. (**D**) Solvent accessible surface area (138–155 nm^2^) showing minimal surface exposure change. (**E**) Intramolecular hydrogen bond count (α-Carotene: ~195–215; orlistat: ~185–210) over the trajectory.

The 100 ns MD simulation was carried out for the pancreatic lipase–alpha-Carotene complex alongside the pancreatic lipase–orlistat complex to compare their dynamic behaviour (Figure 9). RMSD analysis (Figure 9A) showed that both complexes reached equilibrium within the first 15–20 ns and remained stable for the rest of the trajectory. The alpha-Carotene complex maintained RMSD values between 0.15 and 0.22 nm throughout the production run, with a mean of approximately 0.18 nm. The orlistat complex displayed a slightly wider range of fluctuation (0.10–0.25 nm), with brief peaks near 40 ns and 85 ns where values touched 0.24–0.25 nm before settling back. Neither complex showed a persistent upward drift after equilibration, which confirms that both ligands stayed bound and the protein backbone did not undergo major structural rearrangement.

RMSF profiles (Figure 9B) for both complexes followed a similar pattern, with most residues fluctuating below 0.15 nm. Localised peaks appeared near residues 10–30, 50, 100–120, and 260–280, corresponding to surface-exposed loops and terminal regions. The alpha-Carotene complex showed a pronounced peak at the C-terminal region (residue 270–280) reaching approximately 0.40 nm, which was not mirrored in the orlistat complex. This region maps to the lid domain of pancreatic lipase and its higher flexibility may reflect the different steric footprint of alpha-Carotene compared with orlistat in the active site. The core catalytic residues (Ser152, Asp176, His263) remained stable in both complexes.

Radius of gyration (Figure 9C) was steady for both systems, ranging between 1.95 and 2.00 nm. The alpha-Carotene complex held an average Rg of approximately 1.97 nm, while orlistat averaged 1.98 nm. No unfolding or expansion events were detected over the full 100 ns. SASA values (Figure 9D) ranged between 138 and 155 nm-squared for both systems, with the alpha-Carotene complex showing slightly higher mean SASA (around 145 nm-squared) compared with orlistat (around 141 nm-squared). This modest difference may reflect the larger molecular footprint of alpha-Carotene partially protruding from the binding pocket.

Hydrogen bond analysis (Figure 9E) revealed that the alpha-Carotene complex maintained between 195 and 215 intramolecular hydrogen bonds throughout the trajectory, with a mean of approximately 202. The orlistat complex showed a comparable range of 185–210, with a mean close to 195. The slightly higher hydrogen bond count in the alpha-Carotene complex may point to a tighter packing of the protein around the larger ligand, resulting in more protein-protein hydrogen bonds being preserved during the simulation.

### 3.10. Free Energy Landscape and PCA

**Figure 10:**
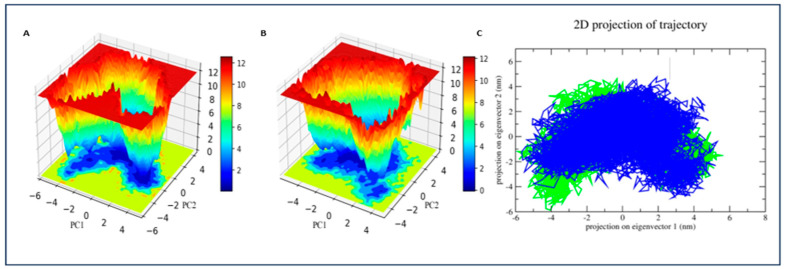
Principal component analysis (PCA) and free energy landscape (FEL) of pancreatic lipase–α-Carotene and pancreatic lipase–orlistat complexes. (**A**) FEL of α-Carotene complex showing a single deep energy basin centred near PC1 ≈ −2 to 0 nm and PC2 ≈ −3 to −1 nm, with minimum ΔG close to 0 kJ/mol and surrounding regions rising to 10–12 kJ/mol, indicating a rigid, thermodynamically stable conformation. (**B**) FEL of orlistat complex showing a broader, more diffuse energy landscape with less sharply defined minima, consistent with greater conformational flexibility. (**C**) 2D projection of MD trajectories along eigenvectors 1 and 2: α-Carotene (blue) forms a dense, compact cluster; orlistat (green) covers a slightly broader region. Neither complex displayed large-scale conformational transitions.

The FEL plots projected onto the first two principal components (PC1 and PC2) are shown in Figure 10. The pancreatic lipase–alpha-Carotene complex (Figure 10A) displayed a well-defined single energy basin centred near PC1 = −2 to 0 nm and PC2 = −3 to −1 nm. The minimum Gibbs free energy in this basin was close to 0 kJ/mol, with surrounding regions rising steeply to 10–12 kJ/mol. The narrow and deep nature of this basin points to a rigid, thermodynamically locked conformation in which the ligand–protein complex spent the majority of the simulation time.

The pancreatic lipase–orlistat complex (Figure 10B) presented a broader and more diffuse energy landscape. While a primary energy minimum was visible, the contours were less sharply defined, and the low-energy region extended over a wider area of the PC1-PC2 plane. This pattern is consistent with greater conformational flexibility in the orlistat-bound state, possibly due to the smaller molecular size of orlistat allowing more movement within the binding pocket. The 2D trajectory projections (Figure 10C) confirmed these observations. The alpha-Carotene trajectory (blue) formed a dense, compact cluster in the centre of the plot, spanning roughly from −4 to +4 nm on eigenvector 1 and −4 to +4 nm on eigenvector 2. The orlistat trajectory (green) covered a slightly broader region, consistent with its wider energy landscape. Neither complex displayed large-scale conformational transitions or bimodal distributions, confirming that both systems remained in a single conformational basin throughout the 100 ns simulation.

### 3.11. MMPBSA Calculation

The binding free energy of the complexes was estimated using the MM-PBSA method from the molecular dynamics trajectories. The α-carotene–pancreatic lipase complex showed a total binding free energy of −89.360 ± 5.367 kJ/mol, whereas the reference inhibitor Orlistat exhibited a stronger binding energy of −150.275 ± 12.863 kJ/mol. Energy decomposition indicated that van der Waals interactions contributed significantly to the binding stability, with values of −114.876 ± 10.171 kJ/mol for α-carotene and −154.458 ± 10.642 kJ/mol for Orlistat. Electrostatic interactions also supported complex formation, with energies of −27.330 ± 15.361 kJ/mol and −55.263 ± 8.365 kJ/mol for α-carotene and Orlistat, respectively. In contrast, polar solvation energy showed positive values (99.223 ± 24.636 kJ/mol for α-carotene and 90.387 ± 10.243 kJ/mol for Orlistat), reflecting the opposing contribution of solvent effects to binding. The non-polar solvation component represented by SASA energy was −13.160 ± 1.004 kJ/mol for α-carotene and −15.983 ± 8.346 kJ/mol for Orlistat, indicating additional stabilisation through hydrophobic interactions, while SAV and WCA energy components were negligible (0.000 kJ/mol) for both complexes. Overall, the results indicate that α-carotene forms a stable complex with pancreatic lipase, with binding mainly driven by van der Waals and hydrophobic interactions, although the binding affinity is lower than that of Orlistat.

## 4. Discussion

Type 2 diabetes mellitus still poses major healthcare problems worldwide, and it is becoming increasingly difficult to prevent and treat it because it is a chronic disease and has many complications. A number of drugs have been developed to control high blood sugar, but the continuous administration of these medications is usually hindered by the occurrence of side effects, and therefore, the therapeutic benefits may diminish [37]. Thus, there is a need for research on novel remedies that are safer and more sustainable for this purpose. Under this framework, scholars are now showing keen interest in the study of natural substances and plant-derived chemicals that exert multiple antioxidant and antihyperglycemic effects [38]. These secondary metabolites derived from medicinal and food plants have been shown to significantly improve metabolic abnormalities and alleviate complications resulting from oxidative stress and insulin resistance [39]. In addition, these products are economically feasible, readily available, and have little or no side effects; hence, patients’ adherence to treatment is expected to be higher when such medicines are prescribed rather than synthetic ones. Then, identifying the cellular mechanisms and therapeutic routes through which the action of nutritionally bioactive phytochemicals leads to protective, pre-diabetic, and diabetic conditions remains important for the development of alternative therapeutic methods and for enhancing clinical outcomes [40,41].

The present study aimed to examine the antidiabetic properties of *Abelmoschus esculentus* (okra) using a combined experimental and computational approach. A key design decision in this revision was to perform molecular docking directly against the four enzymes that were tested in the laboratory assays. This choice was made to establish a coherent link between the in vitro enzyme inhibition data and the computational predictions, rather than to investigate unrelated protein targets. Such an integrated pipeline ensures that docking-derived binding affinities can be interpreted in the context of measured IC50 values and that the two lines of evidence support one another. The enzyme inhibition data showed that the okra fruit extract inhibited all four targets in a concentration-dependent fashion, though the potency was moderate to weak compared with synthetic standards. The fold-difference ranged from 1.49-fold for pancreatic lipase to 22-fold for DPP-4. These numbers need to be viewed in perspective. A crude aqueous extract comprises hundreds of compounds at low individual concentrations, and the observed IC_50_ values represent the combined effect of the entire chemical mixture. Fractionation and isolation of individual compounds would likely yield more concentrated activity. The high IC_50_ values (7–9 mg/mL range for alpha-glucosidase, alpha-amylase, and pancreatic lipase) raise questions about the feasibility of achieving such concentrations in vivo. These levels are difficult to attain solely through dietary intake, and the practical application of the crude extract is limited to a supportive dietary role rather than a standalone therapeutic agent [42].

The molecular docking results provided a structure-level explanation for the enzyme inhibition data. Alpha-Carotene, Vitamin E, and Spiraeoside emerged as the top-ranked compounds across all four enzymes. The strongest binding was observed for alpha-Carotene against pancreatic lipase (−11.1 kcal/mol), which was 4.2 kcal/mol more negative than the positive control orlistat (−6.9 kcal/mol). This finding is consistent with the in vitro data, in which the smallest fold difference between the extract and standard was also observed for pancreatic lipase. The lipophilic nature of alpha-Carotene, a C40 tetraterpenoid with no heteroatoms, allows it to fit deep into the hydrophobic binding cleft of pancreatic lipase, forming extensive van der Waals contacts with Tyr114, Phe77, Phe215, Ile209, and Leu264. These residues line the enzyme’s catalytic pocket, and their engagement by alpha-Carotene may block substrate access to the active site. Spiraeoside, a glycosylated flavonoid, showed a different binding pattern. Its polyphenolic structure enabled multiple hydrogen bonds and electrostatic interactions, making it the most polar binder across all targets. At DPP-4, Spiraeoside formed twelve hydrogen bonds, the highest count for any compound-enzyme pair in this study. This dual character of the okra chemical profile, in which lipophilic terpenoids and polar flavonoids coexist, enables the plant’s extract to bind to both hydrophobic and polar binding sites across different enzymes. Such chemical heterogeneity may account for the broad-spectrum, yet moderate, activity observed in the in vitro assays [43].

The ADME and toxicity profiling of alpha-Carotene returned a generally favourable safety profile with a predicted LD_50_ of 1510 mg/kg (Class 4). The compound showed high gastrointestinal absorption, no blood–brain barrier permeation, and no cytochrome P450 inhibition, all of which are desirable for a dietary compound. The only active toxicity prediction was for mutagenicity (probability 0.71), warranting caution and follow-up with experimental genotoxicity testing. It is worth noting that beta-Carotene, a structural isomer of alpha-Carotene, has been extensively studied in humans and is classified as GRAS (generally recognised as safe) by regulatory agencies, which provides some reassurance, though not a direct equivalence. The 100 ns MD simulation of the pancreatic lipase–alpha-Carotene complex alongside the orlistat control provided evidence that the docking-predicted binding mode is stable under dynamic conditions. Both complexes equilibrated within 20 ns and maintained steady RMSD, Rg, SASA, and hydrogen bond profiles throughout the trajectory. The FEL analysis added a thermodynamic dimension to these kinetic observations. The single, deep energy basin observed for alpha-Carotene points to a well-defined, stable binding conformation, whereas the broader energy landscape of orlistat reflects greater conformational sampling. These results are consistent with the larger molecular footprint of alpha-Carotene, which fills the binding pocket more completely and limits the range of accessible conformations.

Several limitations of this study should be acknowledged. First, the enzyme inhibition assays used a crude aqueous extract rather than purified compounds, which makes it impossible to attribute the observed activity to any single molecule. Second, the docking and MD results are computational predictions and require experimental validation through techniques such as surface plasmon resonance or isothermal titration calorimetry. Third, the in vivo bioavailability of alpha-Carotene from dietary okra consumption has not been established. Fourth, the MD simulation was limited to a single 100 ns run for each complex; replicate simulations or extended timescales would strengthen the confidence in these conclusions. Despite these limitations, the present work provides a coherent molecular framework that links experimental enzyme-inhibition data with structure-based computational analysis of okra phytochemicals targeting the same targets.

## 5. Conclusions

The present study examined the antidiabetic capacity of *Abelmoschus esculentus* (okra) using enzyme inhibition assays, molecular docking against four tested enzymes, PLIP interaction profiling, ADME and toxicity prediction, and a 100 ns molecular dynamics simulation. The okra fruit extract showed moderate-to-weak inhibition of alpha-glucosidase, alpha-amylase, DPP-4, and pancreatic lipase compared with their respective standard drugs. Molecular docking of sixteen drug-like okra phytochemicals identified alpha-Carotene, Vitamin E, and Spiraeoside as the top-ranked compounds. Alpha-Carotene showed the strongest binding affinity (−11.1 kcal/mol) against pancreatic lipase, outperforming the positive control, orlistat, by 4.2 kcal/mol. MD simulation confirmed the stability of this complex over 100 ns, and FEL analysis revealed a single, deep energy basin for the alpha-Carotene-bound state. The ADME profile was generally favourable, though a mutagenicity flag calls for experimental follow-up. These findings position alpha-Carotene as a lead candidate for further in vitro and in vivo validation as an adjunctive agent in diabetes management. Fractionation studies on okra extract and bioavailability assessments of individual phytochemicals are the natural next steps to advance this line of research.

## Figures and Tables

**Table 1:** Inhibitory concentration (IC_50_) values of okra fruit extract and standard inhibitors for α-glucosidase, α-amylase, DPP-4, and pancreatic lipase.

Enzyme	Standard Inhibitor	IC_50_ of Standard (Mean ± SD)	IC_50_ of Okra Extract (Mean ± SD)
α-Glucosidase	Acarbose	3.60 ± 0.12 mg mL^−1^	7.66 ± 0.31 mg mL^−1^
α-Amylase	Acarbose	2.67 ± 0.08 mg mL^−1^	5.21 ± 0.18 mg mL^−1^
DPP-4	Sitagliptin	0.095 ± 0.008 µg mL^−1^	2.11 ± 0.15 µg mL^−1^
Pancreatic lipase	Orlistat	6.15 ± 0.32 mg mL^−1^	9.17 ± 0.54 mg mL^−1^

**Table 2:** Molecular docking of phytocompounds with target proteins.

Compound	Pancreatic Lipase	DPP4	Alpha-Glucosidase	Alpha-Amylase
**Alpha-Carotene**	−11.1	−8.5	−9.2	−9.8
**Vitamin E**	−10.6	−10.2	−8.7	−9.1
**Spiraeoside**	−9.7	−9.2	−8	−8.7
**(+)-Vernolic Acid**	−7	−6.3	−5.8	−5.6
**(1s,2s,3s,6s)-3-ethenyl-3,7,7-trimethyl-2-prop-1-en-2-ylbicyclo[4.1.0]heptane**	−7	−6.4	−5.6	−6.8
**Thiamine**	−6.5	−6.7	−6.3	−5.9
**D-Galactopyranuronic Acid**	−6.4	−6.4	−5.8	−6.4
**Citronellyl Propionate**	−6.4	−5.4	−5.7	−5.1
**D-Glucopyranuronic Acid**	−6.4	−6.4	−5.9	−6.4
**Palmitelaidic Acid**	−6.3	−5.6	−5.1	−5.2
**Myristic Acid**	−6.1	−5.4	−4.9	−4.8
**Undecyl Acetate**	−5.9	−4.5	−4.8	−4.7
**Methyl Linoleate**	−5.9	−5.6	−5.1	−5.1
**D-Glucopyranose**	−5.9	−5.8	−6.2	−6.5
**2-Methylbutyl Heptanoate**	−5.7	−4.9	−4.9	−4.6
**Isoamyl Alcohol**	−3.9	−4.3	−4	−4

**Table 3:** Molecular docking of the targets with standard drugs.

Protein Name	Positive Control	Binding Affinity
**Pancreatic Lipase**	Orlistat	−6.9
**DPP4**	Sitagliptin	−9.1
**Alpha-glucosidase**	Acarbose	−7.2
**Alpha-Amylase**	Miglitol	−5.7

**Table 4:** PLIP interaction profile of selected compounds and positive control with pancreatic lipase (1LPB).

Compound	Residue	Amino Acid	Distance	Interaction
**Alpha-Carotene**	22B	GLU	3.69	Hydrophobic
	77B	PHE	3.66	
	78B	ILE	3.72	
	114B	TYR	3.84	
	114B	TYR	3.44	
	114B	TYR	3.84	
	179B	GLU	3.75	
	180B	PRO	3.48	
	209B	ILE	3.72	
	215B	PHE	3.98	
	256B	ARG	3.7	
	260B	ALA	3.65	
	264B	LEU	3.49	
	264B	LEU	3.69	
**Vitamin E**	77B	PHE	3.99	Hydrophobic
	77B	PHE	3.65	
	114B	TYR	3.83	
	114B	TYR	3.74	
	114B	TYR	3.79	
	114B	TYR	3.64	
	180B	PRO	3.7	
	209B	ILE	3.9	
	209B	ILE	3.79	
	215B	PHE	3.72	
	215B	PHE	3.46	
	259B	ALA	3.87	
	264B	LEU	3.82	
	264B	LEU	3.85	
	256B	ARG	2.94	Hydrogen Bonds
	256B	ARG	2.48	
	256B	ARG	2.78	
**Spiraeoside**	77B	PHE	3.89	Hydrophobic
	215B	PHE	3.69	
	215B	PHE	3.66	
	76B	GLY	2.33	Hydrogen Bonds
	79B	ASP	2.28	
	151B	HIS	2.32	
	152B	SER	2.16	
	256B	ARG	2.67	
	256B	ARG	2.41	
	256B	ARG	2.23	
	114B	TYR	4.01	π-Stacking
	263B	HIS	4.07	Salt Bridges
**(+) Vernolic Acid**	78B	ILE	3.69	Hydrophobic
	114B	TYR	3.65	
	114B	TYR	3.71	
	180B	PRO	3.64	
	215B	PHE	3.66	
	215B	PHE	3.68	
	215B	PHE	3.56	
	215B	PHE	3.79	
	260B	ALA	3.68	
	264B	LEU	3.75	
	76B	GLY	2.61	Hydrogen Bonds
	77B	PHE	2.59	
	151B	HIS	2.47	
	263B	HIS	3.11	
**(1S,2S,3S,6S)-3-ethenyl-3,7,7-trimethyl-2-prop-1-en-2-ylbicyclo[4.1.0]heptane**	77B	PHE	3.28	Hydrophobic
	77B	PHE	3.6	
	114B	TYR	3.61	
	114B	TYR	3.85	
	114B	TYR	3.48	
	153B	LEU	3.98	
	209B	ILE	3.58	
	215B	PHE	3.33	
	215B	PHE	3.26	
**Thiamine**	77B	PHE	3.97	Hydrophobic
	114B	TYR	3.77	
	180B	PRO	3.74	
	215B	PHE	3.92	
	77B	PHE	2.82	Hydrogen Bonds
	77B	PHE	2.57	
	263B	HIS	2.79	
**Positive Control—Orlistat**	78B	ILE	3.74	Hydrophobic
	114B	TYR	3.62	
	114B	TYR	3.75	
	114B	TYR	3.64	
	180B	PRO	3.75	
	209B	ILE	3.81	
	215B	PHE	3.75	
	215B	PHE	3.4	
	215B	PHE	3.56	
	215B	PHE	3.76	
	252B	TRP	3.62	
	255B	THR	3.78	
	256B	ARG	3.78	
	259B	ALA	3.79	
	264B	LEU	3.77	
	76B	GLY	2.52	Hydrogen Bonds
	77B	PHE	2.23	
	151B	HIS	2.41	
	152B	SER	2.13	
	263B	HIS	3.34	
	79B	ASP	4.79	Salt Bridges

**Table 5:** PLIP interaction profile of selected compounds and positive control with DPP-4 (2ONC).

Compound	Residue	Amino Acid	Distance	Interactions
**Vitamin E**	547B	TYR	3.53	Hydrophobic
	547B	TYR	3.64	
	629B	TRP	3.61	
	629B	TRP	3.97	
	629B	TRP	3.57	
	629B	TRP	3.72	
	631B	TYR	3.7	
	631B	TYR	3.65	
	659B	TRP	3.64	
	662B	TYR	3.72	
	711B	VAL	3.97	
	741B	GLY	2.58	Hydrogen Bond
**Spiraeoside**	357B	PHE	3.77	Hydrophobic
	357B	PHE	3.58	
	125B	ARG	2.71	Hydrogen Bond
	206B	GLU	2.79	
	209B	SER	1.79	
	357B	PHE	2.1	
	358B	ARG	2.39	
	358B	ARG	2.81	
	358B	ARG	2.72	
	358B	ARG	2.66	
	361B	GLU	2.82	
	361B	GLU	1.79	
	361B	GLU	2.45	
	666B	TYR	2.43	
	358B	ARG	4.39	Salt Bridge
**Alpha-Carotene**	356B	ARG	3.71	Hydrophobic
	357B	PHE	3.23	
	357B	PHE	3.82	
	547B	TYR	3.8	
	629B	TRP	3.86	
	629B	TRP	3.53	
	629B	TRP	3.67	
	629B	TRP	3.52	
**Thiamine**	357B	PHE	3.83	Hydrophobic
	631B	TYR	3.91	
	631B	TYR	3.48	Hydrogen Bond
	669B	ARG	2.65	
	710B	ASN	3.42	
	205B	GLU	5.4	Salt Bridges
**D-Galactopyranuronic acid**	320B	GLN	2.12	Hydrogen Bond
	352B	GLY	3.12	
	595B	ASN	1.87	
	595B	ASN	2.56	
	596B	ARG	2.46	
	678B	ASP	2.83	
**(1S,2S,3S,6S)-3-ethenyl-3,7,7-trimethyl-2-prop-1-en-2-ylbicyclo[4.1.0]heptane**	240B	PHE	3.5	Hydrophobic
	252B	VAL	3.71	
	252B	VAL	3.74	
**Positive Control—Sitagliptin**	357B	PHE	3.9	Hydrophobic
	357B	PHE	3.75	
	357B	PHE	3.71	
	631B	TYR	3.85	
	662B	TYR	4	
	666B	TYR	3.96	
	666B	TYR	3.73	
	710B	ASN	3.41	Hydrogen Bond
	710B	ASN	3.29	
	207B	VAL	3.72	Halogen Bonds

**Table 6:** PLIP interaction profile of selected compounds and positive control with α-glucosidase (8CB1).

Compound	Residue	Amino Acid	Distance	Interaction
**Alpha-Carotene**	209A	TYR	3.45	Hydrophobic
	211A	VAL	3.64	
	211A	VAL	3.57	
	220A	VAL	3.69	
	220A	VAL	3.6	
	222A	VAL	3.87	
	234A	THR	3.99	
	248A	LEU	3.28	
	248A	LEU	3.53	
	337A	LEU	3.87	
	337A	LEU	3.93	
	341A	ILE	3.87	
	342A	PHE	3.5	
	343A	LEU	3.5	
**Vitamin E**	376A	TRP	3.94	Hydrophobic
	376A	TRP	3.69	
	481A	TRP	3.57	
	481A	TRP	3.66	
	525A	PHE	3.74	
	525A	PHE	3.7	
	525A	PHE	3.71	
	525A	PHE	3.58	
	613A	TRP	3.93	
	649A	PHE	3.51	
**Spiraeoside**	608A	ARG	3.74	Hydrophobic
	355A	LEU	3.5	Hydrogen Bonds
	363A	MET	3.15	
	608A	ARG	2.42	
	608A	ARG	3.07	
	584A	HIS	5.36	Salt Bridges
**Thiamine**	363A	MET	3.89	Hydrophobic
	867A	VAL	3.91	
	717A	HIS	3.3	Hydrogen Bonds
	868A	LEU	2.85	
**D-Glucopyranose**	481A	TRP	2.24	Hydrogen Bonds
	518A	ASP	2.92	
	600A	ARG	2.13	
	613A	TRP	3.46	
	616A	ASP	3.26	
	674A	HIS	2.62	
**D-Glucopyranuronic acid**	404A	ASP	2.99	Hydrogen Bonds
	481A	TRP	2.51	
	600A	ARG	3.43	
	600A	ARG	2.96	
	616A	ASP	2.27	
	674A	HIS	2.31	
	674A	HIS	2.9	
**Positive Control—Acarbose**	594A	ARG	3.62	Hydrophobic
	868A	LEU	3.71	
	868A	LEU	3.8	
	360A	TYR	2.55	Hydrogen Bonds
	363A	MET	2.17	
	608A	ARG	2.32	
	608A	ARG	3.17	
	717A	HIS	2.65	
	717A	HIS	2.54	
	718A	VAL	2.42	
	866A	GLU	2.5	
	869A	GLU	2.76	
	608A	ARG	4.77	Salt Bridges
	717A	HIS	5.4	

**Table 7:** PLIP interaction profile of selected compounds and positive control with α-amylase (5E0F).

Compound	Residue	Amino Acid	Distance	Interaction
**Alpha-Carotene**	49A	VAL	3.82	Hydrophobic
	51A	ILE	3.55	
	52A	TYR	3.84	
	52A	TYR	3.9	
	53A	ASN	3.41	
	59A	TRP	3.78	
	62A	TYR	3.85	
	62A	TYR	3.62	
	106A	ALA	3.74	
	107A	VAL	3.83	
	162A	LEU	3.73	
	162A	LEU	3.9	
	165A	LEU	3.89	
	198A	ALA	3.6	
**Vitamin E**	58A	TRP	3.45	Hydrophobic
	58A	TRP	3.67	
	59A	TRP	3.56	
	59A	TRP	3.68	
	59A	TRP	3.73	
	62A	TYR	3.72	
	165A	LEU	3.66	
	200A	LYS	3.86	
	235A	ILE	3.39	
	63A	GLN	2.66	Hydrogen Bond
**Spiraeoside**	59A	TRP	3.68	Hydrophobic
	59A	TRP	3.92	
	59A	TRP	3.67	
	165A	LEU	3.66	
	165A	LEU	3.75	
	63A	GLN	2.8	Hydrogen Bond
	101A	HIS	3.52	
	197A	ASP	2.47	
	198A	ALA	3.12	
	300A	ASP	2.29	
	305A	HIS	2.74	
**(1S,2S,3S,6S)-3-ethenyl-3,7,7-trimethyl-2-prop-1-en-2-ylbicyclo[4.1.0]heptane**	59A	TRP	3.82	Hydrophobic
	62A	TYR	3.7	
	62A	TYR	3.72	
	62A	TYR	3.36	
	165A	LEU	3.52	
**D-Glucopyranose**	267A	ARG	2.78	Hydrogen Bond
	267A	ARG	2.26	
	301A	ASN	2.48	
	312A	ILE	3.04	
	317A	ASP	2.12	
	346A	ARG	2.42	
	346A	ARG	2.54	
**D-Galactopyranuronic acid**	314A	THR	3.71	Hydrophobic
	267A	ARG	3.12	Hydrogen Bond
	267A	ARG	2.62	
	309A	GLY	2.42	
	310A	ALA	1.91	
	312A	ILE	3.16	
	314A	THR	2.39	
	317A	ASP	2.74	
	346A	ARG	3.01	
	267A	ARG	5.01	Salt Bridges
	346A	ARG	3.66	
**Positive Control—Miglitol**	332A	PRO	3.86	Hydrophobic
	11A	THR	3.55	Hydrogen Bond
	252A	ARG	2.09	
	252A	ARG	2.96	
	289A	SER	2.82	
	332A	PRO	3.58	
	334A	GLY	3.56	
	334A	GLY	3.07	
	336A	THR	3.34	
	398A	ARG	2.15	
	398A	ARG	2.4	
	403A	GLY	3.2	
	421A	ARG	1.95	
	421A	ARG	2.7	

**Table 8:** Physicochemical properties, pharmacokinetic profile, and drug-likeness assessment of α-Carotene.

Physicochemical Properties									
**Formula**	MW	Heavy atoms	Fraction Csp3	Rot Bonds	HA	HD	MR	TPSA	LOGP
**C_40_H_56_**	536.89 g/mol	40	18	10	0	0	184.43	0	12.46
**Pharmacokinetics**									
**Log Kp**	Water solubility	GIA	BBB	P-gp substrate	CYP1A2 inhibitor	CYP2C19 inhibitor	CYP2C9 inhibitor	CYP2D6 inhibitor	CYP3A4 inhibitor
**−9.16**	Soluble	High	No	Yes	No	No	No	No	No
**Drug-likeness**									
**Lipinski**	QEDw	Veber	Egan	Half Life	BA	PAINS	Brenk	Cell Permeability	PAMPA
**2**	0.19	Good	Bad	0	0.55	0	1	−5.19	0.94

**Table 9:** Predicted toxicity profile of α-Carotene from ProTox-II.

Classification	Target	Probability	Prediction
**Organ toxicity**	**Hepatotoxicity**	**0.85**	**Inactive**
	**Nephrotoxicity**	**0.93**	**Inactive**
	**Respiratory toxicity**	**0.75**	**Inactive**
	**Cardiotoxicity**	**0.92**	**Inactive**
**Toxicity endpoints**	**Carcinogenicity**	**0.86**	**Inactive**
	**Immunotoxicity**	**0.73**	**Inactive**
	**Mutagenicity**	**0.71**	**Active**
	**Cytotoxicity**	**0.81**	**Inactive**
	**Clinical toxicity**	**0.71**	**Inactive**
	**Nutritional toxicity**	**0.81**	**Inactive**
**Tox21-Nuclear receptor signalling pathways**	**AhR**	**0.99**	**Inactive**
	**Androgen receptor**	**1**	**Inactive**
	**AR-LBD**	**0.99**	**Inactive**
	**Aromatase**	**0.81**	**Inactive**
	**ER**	**0.95**	**Inactive**
	**ER-LBD**	**0.84**	**Inactive**
	**PPAR-Gamma**	**0.99**	**Inactive**
**Tox21-Stress response pathways**	**Nrf2/ARE**	**0.92**	**Inactive**
	**HSE**	**0.92**	**Inactive**
	**MMP**	**0.84**	**Inactive**
	**P53**	**0.99**	**Inactive**
	**ATAD5**	**0.99**	**Inactive**
**Molecular Initiating Events**	**THRα**	**0.89**	**Inactive**
	**THRβ**	**0.97**	**Inactive**
	**Transtyretrin**	**0.67**	**Inactive**
	**Ryanodine receptor**	**0.93**	**Inactive**
	**GABA receptor**	**0.6**	**Inactive**
	**NMDAR**	**0.84**	**Inactive**
	**AMPAR**	**1**	**Inactive**
	**Kainate receptor**	**1**	**Inactive**
	**Achetylcholinesterase**	**0.8**	**Inactive**
	**NADHOX**	**0.72**	**Inactive**
	**VSGC**	**0.83**	**Inactive**

## Data Availability

The authors declare that all the data supporting the findings of this study are contained within the paper.
